# Optimizing aesthetic results in Asian women with giant phyllodes tumors over 10 cm: the periareolar mastopexy approach

**DOI:** 10.1093/jscr/rjae342

**Published:** 2024-05-28

**Authors:** Kun-Han Chen, Yu-Chen Hsu

**Affiliations:** Department of Plastic Surgery, Ditmanson Medical Foundation Chia-Yi Christian Hospital, No. 539, Zhongxiao Rd., East Dist., Chiayi City 60002, Taiwan; Department of General Surgery, Ditmanson Medical Foundation Chia-Yi Christian Hospital, No. 539, Zhongxiao Rd., East Dist., Chiayi City 60002, Taiwan

**Keywords:** giant phyllodes tumor, breast, periareolar mastopexy

## Abstract

Giant phyllodes tumors, typically exceeding 10 cm in size, are neoplastic lesions with malignant potential. Surgical excision in small-breasted Asian women presents unique challenges where expected poor aesthetic outcomes may delay timely medical intervention. The periareolar mastopexy technique offers a comprehensive solution, enabling complete tumor removal alongside mastopexy to achieve optimal breast contouring. This approach consistently delivers favorable aesthetic outcomes, enhancing symmetry and contour. Additionally, the periareolar approach minimizes visible scarring, thereby enhancing patient satisfaction with the cosmetic outcome. Herein, we present a case report of Asian women with giant phyllodes tumors exceeding 10 cm, successfully managed using the periareolar mastopexy technique, emphasizing the importance of optimizing aesthetic outcomes in these challenging cases.

## Introduction

Giant phyllodes tumors, a rare type of fibroepithelial breast neoplasms, are characterized by their significant size, often exceeding 10 cm in diameter. These tumors present a considerable challenge in clinical management due to their potential for rapid growth and tendency to recur locally. Phyllodes tumors have distinct histological features, including stromal overgrowth and leaf-like projections, covering a range from benign to malignant behavior. However, the primary concern with giant phyllodes tumors is their potential for malignancy [1].

Surgical excision is the standard treatment, aiming to completely remove the tumor while minimizing the risk of recurrence. However, determining the best surgical approach, especially in small-breasted Asian women, poses unique challenges. Concerns about anticipated poor aesthetic outcomes may lead to delays in seeking medical care. These challenges go beyond oncological aspects and also include cosmetic outcomes. This case report provides a comprehensive overview of managing giant phyllodes tumors, emphasizing the complexities of diagnosis and treatment, with a focus on optimizing both oncological and aesthetic outcomes.

## Case presentation

A 50-year-old female presented to the outpatient Breast Clinic with a complaint of a painless lump in her right breast persisting for four years, progressively increasing in size. She was a non-smoker with no significant family history of breast disease. The patient sought medical attention 10 months prior to this visit when her tumor was ~10 cm in size as noted on mammography. A core needle biopsy revealed a benign breast lesion. However, she was lost to follow-up due to concerns about potential unfavorable cosmetic outcomes. Subsequently, the tumor progressed to a size comparable to that of a melon, exhibiting a lobulated contour, prompting her decision to pursue treatment.

**Figure 1:**
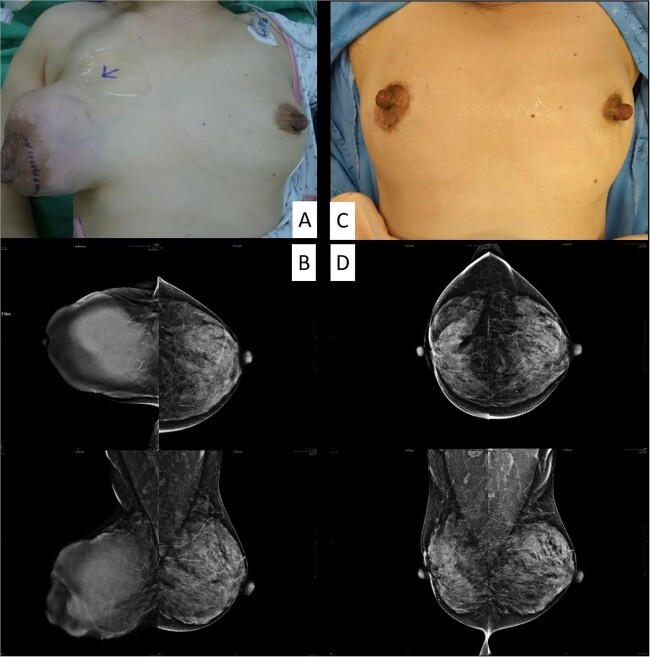
The clinical picture and mammography before (A, B) and after (C, D) the operation.

**Figure 2:**
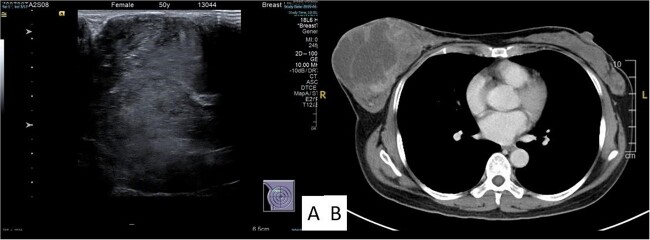
Ultrasound (A) and computed tomography (B) image of the tumor.

Examination revealed a firm, lobulated mass, measuring ~12 × 14 cm, predominantly occupying the breast, with the overlying skin remaining unaffected (Fig. 1A). No signs of skin ulceration, nipple inversion, discharge, or enlarged axillary lymph nodes were observed. Mammography indicated a well-defined, high-density lobular tumor measuring 12 cm, lacking any of calcifications (Fig. 1B). Breast ultrasound revealed a well-defined homogenous hypoechoic mass lesion larger than 10 cm (Fig. 2A). CT scan confirmed a heterogeneous soft tissue lesion within the breast, with no evident involvement of the chest wall, axillary lymph nodes, or distant metastasis (Fig. 2B).

**Figure 3:**
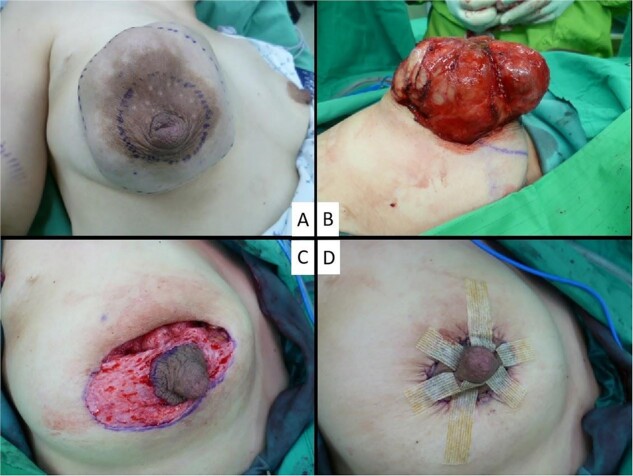
Periareolar mastopexy approach for giant phylloedes tumor.

Wide local tumor excision by periareolar mastopexy approach was performed to resect the tumor and restore breast symmetry. Pathology report showed a 14-cm borderline phyllodes tumor. Cosmetic outcome was evaluated by the breast and plastic surgeon showed favorable aesthetic results as the treated breast was slightly different than the untreated. Minimal postoperative scarring was observed over the new areolar complex (Fig. 1C). Serial breast image follow-up, including mammography and breast echo, showed no evidence of tumor local recurrence for 3 years (Fig. 1D).

## Discussion

A patient facing a breast tumor over 10 cm often experiences significant physical discomfort and emotional distress. The size of the tumor implies that it may have been growing for a long time, possibly reaching an advanced stage. It is common for these patients to fear negative treatment outcomes, which can lead to delays in seeking timely care. In such situations, having a surgeon who can offer a positive cosmetic outcome becomes essential in encouraging patients to accept and pursue treatment actively. The periareolar mastopexy approach proves to be a valuable surgical strategy in this context, offering wide surgical exposure. It enables the excision of benign giant breast tumors while concurrently restoring breast shape, resulting in favorable aesthetic outcomes and minimal postoperative scarring [2–4].

The procedure begins by marking a pair of concentric circumareolar skin incisions (Fig. 3A). The inner circle’s diameter is adjusted to match the areolar diameter and ensure symmetry with the opposite breast. Meanwhile, the outer circle’s diameter is determined using four standard reference points: upper, lower, inner, and outer. These points are connected to form a circular pattern. The distances from the upper point to the sternal notch, the lower point to the inframammary fold, the inner point to the sternal notch, and the outer point to the anterior axillary line all corresponded to their respective distances from the nipple in the unaffected contralateral breast.

Initially, a preplanned full-thickness incision is made along approximately one-third to half of the circumference of the outer periareolar circle, targeting the area near the apex of the tumor. This approach ensures easy access to the phyllodes tumor with a clear and optimal view, facilitating its resection with an adequate safety margin (Fig. 3B). Following the wide local excision of the tumor, deepithelialization of the entire skin between the inner and outer periareolar incisions was performed, ensuring that no dermoglandular tissue was discarded (Fig. 3C). The expanded excessive skin envelope could be mobilized to achieve restoration of breast symmetry. The breast parenchyma was carefully repositioned during the process of approximating the new nipple. For skin closure, a round block suture technique is employed at the outer skin margin to reduce its diameter to that of the normal areola, resulting in only a periareolar scar (Fig. 3D).

Surgical excision remains the primary treatment for giant phyllodes tumors, but it is vital to prioritize aesthetic outcomes, which significantly impact body image and quality of life. The periareolar mastopexy approach is particularly recommended when there is no skin or nipple-areolar complex ulceration due to the tumor. This technique allows extensive surgical exposure for excising benign giant breast tumors while restoring breast shape, leading to favorable aesthetic results with minimal periareolar scarring [5]. The adoption of the periareolar mastopexy approach offers an enhanced treatment option for patients concerned about poor expected surgical outcomes, especially those facing tumors exceeding 10 cm in size. This approach ensures comprehensive care and improved outcomes tailored to individual needs.
